# Evaluating neonatal mortality in Malta compared with other EU countries: Exploring the influence of congenital anomalies and maternal risk factors

**DOI:** 10.1111/ppe.13106

**Published:** 2024-09-06

**Authors:** Merle Wilhelm, Miriam Gatt, Rok Hrzic, Neville Calleja, Hajo Zeeb

**Affiliations:** ^1^ University of Maastricht Maastricht The Netherlands; ^2^ Malta Congenital Anomalies Registry, Directorate for Health Information and Research Pietà Malta; ^3^ Department of International Health, Care and Public Health Research Institute Maastricht University Maastricht The Netherlands; ^4^ Directorate for Health Information and Research Pietà Malta; ^5^ Leibniz Institute for Prevention Research and Epidemiology‐BIPS Bremen Germany

**Keywords:** anomalies, birth defects, maternal age, maternal education, maternal nationality in Malta, neonatal mortality rate

## Abstract

**Background:**

Globally, 240,000 babies die in the neonatal period annually due to congenital anomalies (CA). Malta reports the highest neonatal mortality rate (NMR) among EU (European Union) Countries, constituting a public health concern.

**Objectives:**

This study describes the contribution of CA to NMR in Malta, investigating possible associations with known maternal risk factors of maternal age, nationality, and education. Additionally, it provides an update on the contribution of CA to neonatal deaths in Malta and other EU countries.

**Methods:**

Anonymous data for births and neonatal deaths were obtained for 2006–2020 from the National Obstetrics Information System (NOIS) in Malta. Regression analyses adjusting for maternal risk factors were run on this data to explore possible associations with NMR. NMRs published by EUROSTAT 2011–2020 were used to compare mortality by underlying cause of death (CA or non‐CA causes) for Malta and other EU countries.

**Results:**

Between 2006 and 2020, 63,890 live births with 283 neonatal deaths were registered in Malta, (NMR 4.4 per 1000 live births). CA accounted for 39.6% of neonatal deaths. No time trends were observed in either total NMR, NMR attributed to CA or mortality due to non‐CA causes. Adjusted variables revealed associations for women hailing from non‐EU, low‐income countries. Malta registered high NMRs compared to EU countries, most marked for deaths attributed to CA.

**Conclusions:**

Between 2006 and 2020, Malta's NMR remained stable. Maternal Nationality, from non‐EU low‐income countries, was associated with higher neonatal mortality. The influx of such migrants may play a partial role in the high NMRs experienced. Malta's high NMR was primarily driven by early neonatal deaths, which included high proportions of deaths due to CA and is linked to the fact that termination of pregnancy is illegal in Malta.

**Synopsis**

**Study question**

What is the contribution of congenital anomalies (CA) to Neonatal Mortality Rate (NMR) in Malta? Is there an association between NMR and maternal age, nationality, education in Malta? How does the contribution of CA to NMR compare to other EU (European Union) countries?

**What is already known**

Malta has the highest reported NMR in the European Union (EU). The latest national study published in 2015 showed this rate was strongly influenced by high mortality rates among newborns with CA.

**What the study adds**

This study analyses CA contributions to NMR in Malta. When adjusted for maternal age, nationality and education, only maternal nationality emerges as associated with increased NMR, particularly for non‐EU women. The study confirms that CA negatively affects NMR and it is important to consider country policies and uptake of termination of pregnancy when comparing NMRs between countries.

## 1 BACKGROUND

In 2021, around 2.3 million babies died within their first 28 days of life.^1^ Neonatal mortality encompasses deaths until 28 days regardless of gestational age at live birth.^1^, ^2^ The Sustainable Development Goal (SDG) 3.2.2, calls for reducing neonatal mortality to 12 deaths per 1000 live births by 2030.^2^ In 2021, the global neonatal mortality rate (NMR) was reported as 18 deaths per 1000 live births.^3^ Decreasing NMR is evident in Europe, where NMR has declined by 78% from 1990 to 2021, with an NMR of 2 per 1000 live births reported in 2021.^1^ However, countries within Europe differ widely in reported NMRs. In 2019, Iceland reported the lowest NMR of 0.5 per 1000 live births, while Malta reported the highest NMR of 4.3 per 1000 live births.^4^

NMR is an important indicator for measuring a country's social and economic development, overall health status and accessibility of healthcare.^5^ Neonatal mortality may be attributed to either non‐congenital anomaly causes or congenital anomaly (CA) causes.^6^ Analysing the causes contributing to neonatal mortality is crucial for identifying potentially modifiable healthcare issues and socioeconomic drivers.^5^ Maternal characteristics, predominantly maternal age, education and nationality can influence NMRs.^7^, ^8^, ^9^ Older maternal age, lower education and immigrant maternal status are known to increase adverse pregnancy outcomes as they are linked to inadequate perinatal care and disparities in perinatal health services.^7^, ^8^, ^9^

Non‐CA causes of NMR, namely prematurity and birth asphyxia, have been seen to decrease as a result of advances in perinatal healthcare.^10^, ^11^ However, the causes of CA remain mainly unknown with no effective prevention or treatment such that their occurrence has not decreased to such an extent.^6^ Decreasing NMRs attributed to CA in high‐income countries has been related to higher rates of TOPFA (termination of pregnancy due to fetal anomaly).^12^

CA are recognised by WHO as one of the main causes of global burden of disease, with 240,000 neonatal deaths due to CA occurring worldwide annually.^10^ A study by Gatt et al.^13^ in Malta, analysing data between 1994 and 2013, observed that non‐CA causes of death were decreasing, while mortality attributed to CA causes remained stable over the 20‐year period. Malta is the only EU (European Union) country where TOPFA is illegal and has the highest reported NMR in Europe, this is in part due to the inclusion of babies with CA, who, in other countries, would not all be delivered.^10^, ^13^ The European Perinatal Health Report, 2015–2019 (2022), also shows Malta reporting comparatively high rates of early neonatal mortality, with a rate of 3.6 deaths per 1000 live births in 2019, while late neonatal mortality was comparable to other countries at 0.7 per 1000 live births.^4^

This study aims to, describe and analyse Malta NMR adjusting for maternal age, nationality and education to understand the contribution of these factors to NMR.^7^, ^8^, ^9^ Furthermore, it updates the last analysis describing the contribution of CA to NMR in Malta compared to other EU countries.^13^ This study utilises a longer time period and enables understanding of the current situation.

## 2 METHODS

Anonymised data for all live births and neonatal deaths in Malta were obtained from the National Obstetrics Information System (NOIS) of the Directorate for Health Information and Research for the 15‐year period 2006–2020. This national register collects information on all births and includes neonatal deaths occurring from 22‐week gestation. NMRs were calculated by dividing number of neonatal deaths by the total number of live births within the same time frame.^14^

### 2.1 Statistical analysis

Time trends and proportions of deaths by cause were analysed with Poisson regression using Statistical Package for the Social Sciences (SPSS). In order to allow for a potential break in series due to a rapid rise in migration rates during the timeframe, a binary variable denoting whether the timeframe was pre‐ or post‐2011 was used. This approach was done in concordance with the migration influx from 2011 onwards and aimed to address any potential disparities resulting from shifts in population movement.^15^ Logistic regression analyses were conducted and the calculation of expectation–maximisation means was added to facilitate interpretation.^14^

This analysis further adjusted the Maltese data for potential confounders known to be associated with neonatal mortality, including maternal age (“<19”, “20–35”, “>35”), nationality (“Maltese”, “Pre2004 EU”, “Post2004 EU”, “NE (non‐EU) high income”, “NE upper middle income”, “NE lower middle income”, “NE low income”) and education (“tertiary education” and “non tertiary education”). Nationality served as an indicator for immigrant women, whereas education was selected as a marker for the mother's socioeconomic status.^8^, ^9^

To compare NMRs attributable to CA and non‐CA causes in Malta and other EU countries, the longest available data series from EUROSTAT website was extracted for 2011–2020. Data for neonatal mortality was analysed and compared for three main categories: (i) all causes of death, (ii) those attributed to non‐CA causes and (iii) those due to CA causes. Data was classified into early (0–6 days) and late neonatal death (7–28 days).^2^ Underlying causes of death coded within the ICD 10 ‘Q‐chapter’ were taken as deaths attributed to CA. All other codes were grouped as non‐CA causes.^16^

### 2.2 Missing data

Within the Maltese dataset used for potential confounders, the variable with most missing data was “education” with an overall 22% missing data. Maternal age had 0.0% missing data while nationality had 0.3% missing data. Infant outcome was available for all births.

Data on education was mainly missing for non‐EU citizens, born in low‐income countries (31.9%). Maltese citizens on the other hand had 21.6% of the information on educational level missing, while citizens of the countries joining the EU before 2004 had 17.9% missing data. It was assumed that the missing data for education were missing at random.

When comparing EUROSTAT data for NMRs in EU countries, Estonia, Luxembourg, Cyprus, and Slovenia were excluded as these data were not available from EUROSTAT due to the lack of data and/or confidentiality of data whereby exact numbers are not published due to very small numbers. Data from the UK was included from 2011 to 2018, as data from 2018 onwards were unavailable. Where Malta data was missing in the EUROSTAT database due to confidentiality issues arising from small numbers, exact figures were obtained from the NOIS. 95% confidence interval rates were used when analysing and presenting the NMR in Malta in comparison to the EU average rate.

### 2.3 Ethics approval

This study utilised anonymised, non‐identifiable registry data prepared in accordance with General Data Protection Regulation (GDPR) requirements and EUROSTAT published data. Ethical review was not required because the research involved secondary use of existing anonymised registry data and did not involve direct interaction with human subjects.

## 3 RESULTS

### 3.1 Neonatal mortality in Malta

**FIGURE 1:**
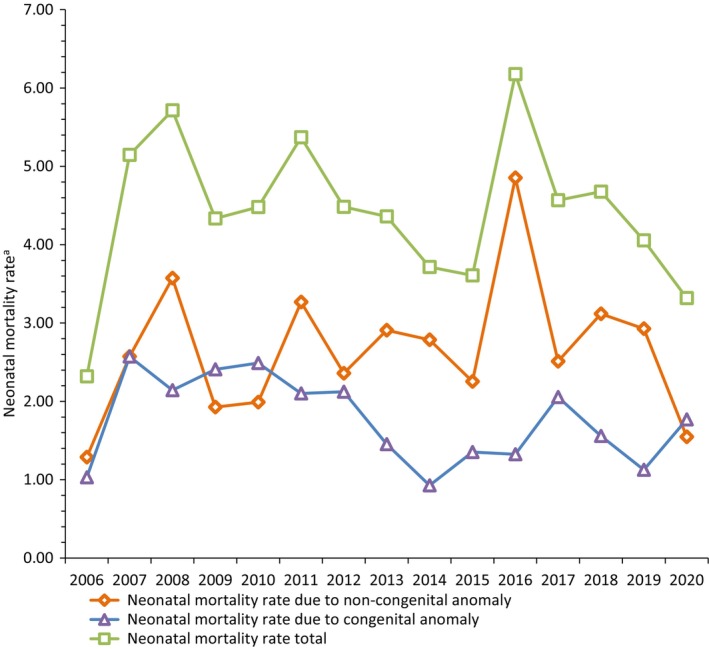
Malta 2006–2020‐NMR by attributed underlying cause. ^a^Neonatal Mortality Rate per 1000 live births. Data were taken from National Obstetrics Information System.

Between 2006 and 2020, a total of 283 neonatal deaths were registered out of 63,890 live births in Malta, with an NMR of 4.4 per 1000 live births (95% CI 3.91, 4.94). The NMR remained relatively stable during this period, ranging from 2.3 to 6.2 per 1000 live births (Figure 1). Poisson's regression for trend showed that the confidence interval includes 1 (odds ratio 0.99, 95% CI 0.97, 1.02). Of the 283 neonatal deaths, 39.6% (*n* = 112) were attributed to CA causes, while the remaining 60.4% (*n* = 171) were due to non‐CA causes. The main CA‐related causes were Anencephaly, Multiple congenital malformations, and Hypoplastic left heart syndrome. The proportion of neonatal deaths attributed to CA causes fluctuated between 55.6% in 2009 and 21.4% in 2016 (Figure 1). No trend was noted over time with confidence interval encompassing the value of 1 (odds ratio 0.97, 95% CI 0.93, 1.01). Similar results were obtained from the analysis of NMR attributed to non‐CA causes, (odds ratio 1.01, 95% CI 0.98, 1.05). The majority of neonatal deaths were attributed to non‐CA‐related deaths and rates ranged from 44.4% in 2009 to 78.6% in 2016. Despite the evident break in series in reported migration rates for Malta, this did not have an association with mortality rates at any of the time points tested.

### 3.2 The influence of maternal education, nationality, and age on NMR in Malta

Anonymous Malta data from NOIS for the 15‐year period 2006 to 2020 was obtained for all births by maternal age, nationality, educational level and infant outcome. This data was used to analyse infant outcomes adjusted for the above variables. Descriptive statistics showed variability in maternal nationality, with 16.4% of mothers giving birth in Malta over the time period being foreign residents (Table S1). Of these, 62% were of non‐EU nationality, namely Syria (668 mothers), Somalia (453) and Eritrea (322).

Logistic regression analysis indicated that nationality was associated with the odds of neonatal mortality. In particular, mothers from non‐EU, low‐income countries had 2.23 times higher odds of neonatal mortality compared to Maltese women (95% CI 1.34, 3.71). Overall, around 1% of births to low‐income non‐EU women resulted in neonatal mortality (Table S2). Furthermore, there was no difference in proportion of neonatal deaths due to CA between the whole population (39.8% due to CA) and women hailing from non‐EU, low‐income countries (35.7% due to CA). The overall NMR showed no trend when considering only mothers of Maltese nationality or mothers of foreign nationality.

### 3.3 Neonatal mortality comparison with other EU countries

Using data extracted from EUROSTAT database for 2011 to 2020, overall total NMR in Malta was reported as 4.5 per 1000 live births, while the average EU NMR was 2.5 per 1000 live births. Malta's reported NMR was comparatively high, with similar rates observed in Romania (4.1 per 1000 live births) and Bulgaria (4.0 per 1000 live births), while Finland had the lowest reported NMR (1.4 per 1000 live births) (Table 1).

**TABLE 1:** Neonatal Mortality Rate with 95% CI due to congenital anomaly and non‐congenital anomaly causes from 2011 to 2020.

Countries	Total Live Births	Number of neonatal deaths	Neonatal Mortality Rate (95% CI)	Neonatal Mortality Rate CA‐causes (per 1000 live births)	Neonatal Mortality Rate non‐CA‐causes (per 1000 live births)
EU^a^	43,073,021	106,144	2.46 (2.45, 2.48)	0.6	1.9
Malta	42,846	193	4.50 (3.87, 5.14)	1.6	2.9
Austria	831,892	1958	2.35 (2.25, 2.46)	0.7	1.6
Belgium	1,221,600	2843	2.33 (2.24, 2.41)	0.6	1.7
Bulgaria	651,840	2612	4.01 (3.85, 4.16)	0.7	3.3
Croatia	382,994	1204	3.14 (2.97, 3.32)	0.9	2.3
Czechia	1,108,159	1827	1.65 (1.57, 1.72)	0.3	1.3
Denmark	594,453	1624	2.73 (2.60, 2.86)	0.5	2.3
Finland	533,080	743	1.39 (1.29, 1.49)	0.5	0.9
France	7,880,801	20,360	2.58 (2.55, 2.62)	0.5	2.1
Germany^b^	7,386,599	17,038	2.31 (2.27, 2.34)	0.6	1.7
Greece	921,347	2253	2.45 (2.34, 2.55)	0.6	1.8
Hungary	923,639	2393	2.59 (2.49, 2.69)	0.6	2.0
Ireland	649,427	1563	2.41 (2.29, 2.53)	1.0	1.4
Italy	4,779,767	9988	2.09 (2.05, 2.13)	0.4	1.7
Latvia	201,491	578	2.87 (2.64, 3.10)	0.6	2.3
Lithuania	292,461	665	2.27 (2.10, 2.45)	0.8	1.5
Netherlands	1,722,293	4798	2.79 (2.71, 2.86)	0.7	2.1
Poland	3,791,397	11,109	2.93 (2.88, 2.98)	0.9	2.0
Portugal	868,759	1804	2.08 (1.98, 2.17)	0.4	1.7
Romania	2,011,355	8334	4.14 (4.05, 4.23)	1.0	3.2
Portugal	868,759	1804	2.08 (1.98, 2.17)	0.4	1.7
Slovakia	568,675	1750	3.08 (2.9, 3.22)	1.0	2.1
Spain	4,063,057	7943	1.95 (1.91, 2.00)	0.4	1.6
Sweden	1144,590	1815	1.59 (1.51, 1.66)	0.4	1.2
UK^c^	6,924,515	17,133	2.47 (2.44, 2.51)	0.6	1.9

^a^ Re Countries from 2020.

^b^ Germany: (until 1990 former territory of the FRG).

^c^ UK includes data until 2018. Data taken from EUROSTAT. https://ec.europa.eu/eurostat/databrowser/view/HLTH_CD_AINFO__custom_5642800/default/table?lang=en (Accessed June 26, 2023).

**FIGURE 2:**
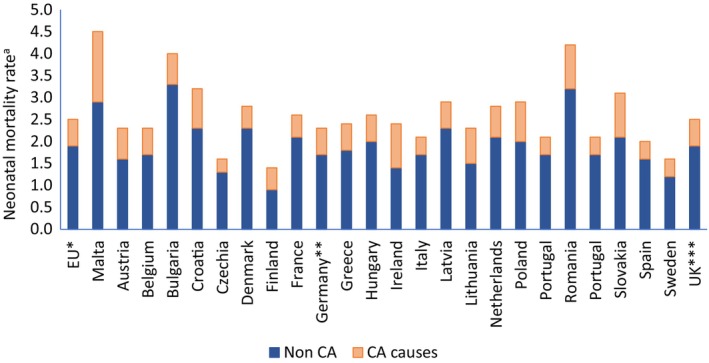
NMR by country and underlying cause of death: Malta versus EU Countries (2011–2020). ^a^Neonatal Mortality Rate per 1000 live births. ^b^EU Countries from 2020, ^c^Germany: (until 1990 former territory of the FRG), ^d^UK includes data until 2018. Based on Table 1. Data taken from EUROSTAT. https://ec.europa.eu/eurostat/databrowser/view/HLTH_CD_AINFO__custom_5642800/default/table?lang=en (Accessed June 26, 2023).

**FIGURE 3:**
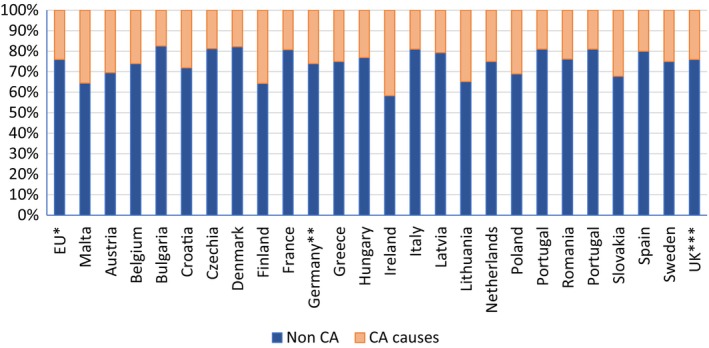
Proportion of CA‐related neonatal mortality to non‐CA‐related mortality: Malta versus EU Countries (2011–2020). ^a^EU Countries from 2020, ^b^Germany: (until 1990 former territory of the FRG), ^c^UK includes data until 2018. Based on Table 1. Data taken from EUROSTAT. https://ec.europa.eu/eurostat/databrowser/view/HLTH_CD_AINFO__custom_5642800/default/table?lang=en (Accessed June 26, 2023).

Malta had the highest NMR attributed to CA among EU nations with the relative proportion of NMR due to CAs being similar to that in Ireland where TOPFA has been illegal until 2019 (Figures 2 and 3).

**FIGURE 4:**
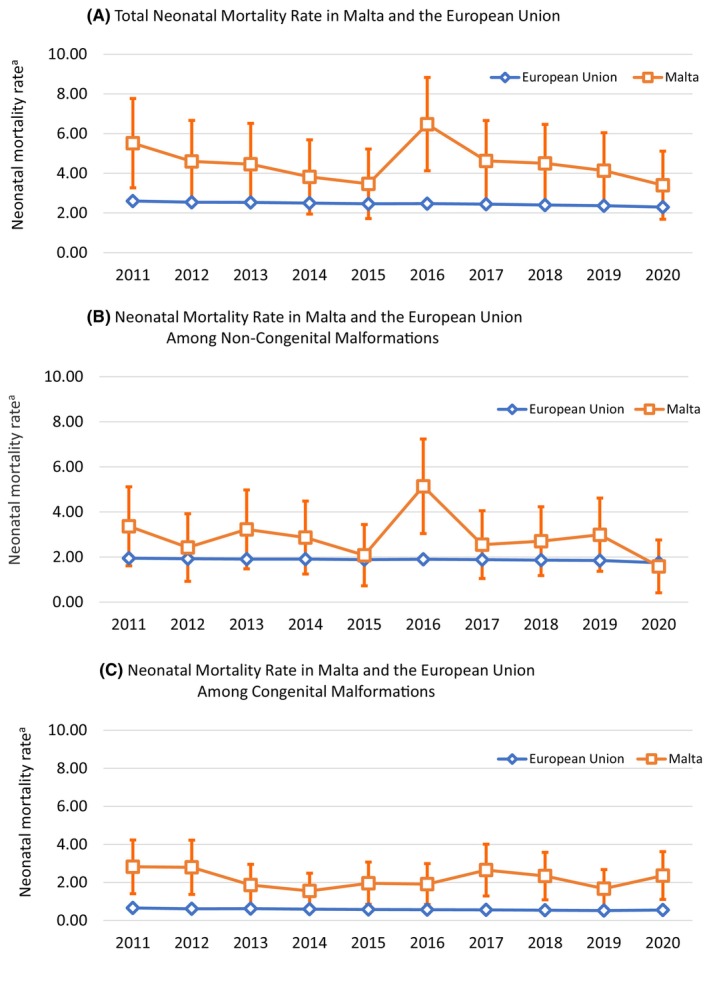
Examining NMR Trends in Malta (2011–2020): Error Bars and comparisons with EU Average. (A) Total NMR in Malta versus EU, (B) NMR due to non‐congenital anomaly causes in Malta versus EU, (C) NMR due to congenital anomaly causes in Malta versus EU. ^a^Neonatal Mortality Rate per 1000 live births. Data taken from EUROSTAT.

When considering deaths due to non‐CA causes, Malta's NMR aligned more closely with other EU countries (Table 1). A comparative analysis of the total NMR in Malta versus the EU average from 2011 to 2020 revealed that the 95% confidence interval for Malta included the EU average in all years except for 2011 and 2016 (Figure 4A). In Figure 4B, showing NMR due to non‐CA causes, Malta was found to be higher only in 2016 when compared to the EU average, while in all other years, the confidence intervals included the EU average. Conversely, Figure 4C shows that the NMR due to CA causes was consistently higher than EU average throughout the time period.

The comparatively higher NMRs were particularly prominent during the first 6 days of life and even more prominent when comparing the first day of life (very early neonatal period). Higher rates were observed, when comparing both total NMRs and NMRs due to CA and non‐CA causes (Table 2). In Malta, between 2011 and 2020, 52% of neonatal deaths caused by CA occurred within the first day of life, while the EU average for the same period was 39%. When focusing on total neonatal deaths unrelated to CA causes, Malta's mortality rate (2.9 per 1000 live births) aligned more closely with the average EU NMR (1.9 per 1000 live births). 81% of neonatal deaths attributed to non‐CA causes in Malta occurred within the first 6 days, compared to the EU average of 73% (Table 2).

**TABLE 2:** Neonatal Mortality Rate by attributed underlying cause during the first and 1–6 days from 2011 to 2020.

Countries	Total Live Births	Neonatal Mortality Rate 1–6 days							
		Number of neonatal deaths	Neonatal mortality rate (95% CI)^a^	Neonatal Mortality Rate CA‐causes/1000 LB	Neonatal Mortality Rate non‐CA‐causes/1000 LB	Number of neonatal deaths	Neonatal mortality rate (95% CI)^a^	Neonatal Mortality Rate CA‐causes/1000 LB	Neonatal Mortality Rate non‐CA causes/1000 LB
EU^b^	43,073,021	41,515	1.0 (0.99, 1.01)	0.2	0.7	34,836	0.8 (0.80, 0.82)	0.2	0.6
Malta	42,846	108	2.5 (2.03, 2.97)	0.8	1.7	49	1.1 (0.82, 1.46)	0.5	0.7
Austria	831,892	1144	1.4 (1.32, 1.48)	0.5	0.9	450	0.5 (0.49, 0.59)	0.1	0.4
Belgium	1,221,600	1111	0.9 (0.85, 0.95)	0.2	0.7	1001	0.8 (0.77, 0.87)	0.2	0.6
Bulgaria	651,840	816	1.3 (0.85, 0.95)	0.2	1.0	1021	1.6 (1.47, 1.66)	0.2	1.3
Croatia	382,994	476	1.2	0.3	0.9	418	1.1	0.3	0.8
Czechia	1,108,159	518	0.5 (0.46, 0.54)	0.1	0.4	717	0.6 (0.60, 0.69)	0.1	0.5
Denmark	594,453	940	1.6 (0.46, 0.54)	0.3	1.3	469	0.8 (0.72, 0.86)	0.1	0.7
Finland	533,080	313	0.6 (0.53, 0.67)	0.2	0.4	274	0.5 (0.45, 0.57)	0.2	0.4
France	7,880,801	7348	0.9 (0.88, 0.92)	0.1	0.8	6773	0.9 (0.84, 0.88)	0.2	0.7
Germany^c^	7,386,599	9333	1.3 (1.27, 1.33)	0.3	1.0	3960	0.5 (0.52, 0.55)	0.1	0.4
Greece	921,347	641	0.7 (1.12, 1.28)	0.2	0.5	868	0.9 (0.88, 1.00)	0.2	0.7
Hungary	923,639	690	0.7 (0.65, 0.75)	0.2	0.6	874	0.9 (0.88, 1.01)	0.2	0.7
Ireland	649,427	751	1.2 (1.12, 1.28)	0.4	0.7	474	0.7 (0.66, 0.80)	0.3	0.4
Italy	4,779,767	3505	0.7 (0.68, 0.72)	0.1	0.6	3461	0.7 (0.70, 0.75)	0.1	0.6
Latvia	201,491	411	2.0 (1.80, 2.20)	0.4	1.7	32	0.2 (0.10, 0.21)	0.0	0.1
Lithuania	292,461	250	0.9 (0.79, 1.01)	0.3	0.6	200	0.7 (0.59, 0.78)	0.3	0.4
Netherlands	1,722,293	2080	1.2 (1.15, 1.25)	0.3	0.9	1546	0.9 (0.85, 0.94)	0.2	0.7
Poland	3,791,397	4847	1.3 (1.26, 1.34)	0.4	0.8	3297	0.9 (0.84, 0.90)	0.2	0.6
Portugal	868,759	579	0.7 (0.64, 0.76)	0.1	0.5	628	0.7 (0.67, 0.78)	0.1	0.6
Romania	2,011,355	1605	0.8 (0.76, 0.84)	0.2	0.6	4022	2.0 (1.94, 2.069)	0.4	1.6
Slovakia	568,675	565	1.0 (0.92, 1.08)	0.4	0.6	578	1.0 (0.93, 1.10)	0.3	0.7
Spain	4,063,057	2523	0.6 (0.58, 0.62)	0.1	0.5	2816	0.7 (0.67, 0.72)	0.1	0.6
Sweden	1144,590	636	0.6 (0.56, 0.64)	0.1	0.4	676	0.6 (0.55, 0.64)	0.1	0.5
UK^d^	6,924,515	8338	1.2 (1.17, 1.23)	0.3	0.9	4962	0.7 (0.70, 0.74)	0.2	0.5

Abbreviation: LB, live births.

^a^ The rates are per 1000 live births.

^b^ EU Countries from 2020.

^c^ Germany: (until 1990 former territory of the FRG).

^d^ UK includes data until 2018. Data taken from EUROSTAT. https://ec.europa.eu/eurostat/databrowser/view/HLTH_CD_AINFO__custom_5642800/default/table?lang=en (Accessed June 26, 2023).

## 4 COMMENT

### 4.1 Principal findings

Between 2006 and 2020, the NMR in Malta was 4.4 per 1000 live births, with 39.6% of the 283 neonatal deaths attributed to CA causes and 60.4% attributed to non‐CA causes. After adjusting the data for maternal age, nationality and education, only maternal nationality, especially for women from non‐EU, low‐income countries, was associated with a higher relative risk of neonatal mortality. Compared with other EU countries, Malta exhibited the highest reported NMR for overall total NMR and neonatal mortality attributed to CA causes. However, the rate for neonatal mortality due to non‐CA causes was not different to the EU average.

### 4.2 Strengths of the study

Strengths of this study include the utilisation of national official statistics with a comprehensive coverage of the islands of Malta and Gozo. Another strength was the availability of national data for adjustment for the variables maternal education, age and nationality, enabling the study to identify potential correlations and areas of challenge. The EU analysis represents a robust strategy for comparing mortality rates between countries exploring EUROSTAT data with and without CA.

### 4.3 Limitations of the data

The study's reliance on registry data, while convenient and appropriate for broad trends, may lack the nuanced detail required to fully understand individual case complexities. The method of attributing deaths to CA based on underlying causes of death coded within the ICD 10 ‘Q‐chapter’, has been demonstrated to underestimate the deaths due to CA according to research by Rissman et al.,^17^ suggesting that CA likely account for an even larger proportion of deaths.

The wide discrepancy between the NMR due to non‐CA mortality rate in 2009 (44.4%) and 2016 (78.6%) may be attributed to the small population size in Malta, resulting in small number variation.^18^

Further limitations of this study included the exclusion of four countries due to missing data in the EUROSTAT database, leading to an incomplete comparison between all EU countries. The Maltese data could not be adjusted for all maternal factors known to possibly be associated with NMR such as obesity, environmental exposures or delayed breastfeeding as these variables were not available.^19^ Non‐maternal variables potentially affecting NMR e.g. infant gender, gestational age and healthcare practices were also not analysed as they were beyond the scope of the study.^20^ Lastly, the existence of missing data on the covariates analysed may lead to bias and incomplete understanding of their association.

### 4.4 Interpretation

In contrast to a previous publication by Gatt et al.^13^ analysing Malta data from 1994 to 2013, no time trends were observed in overall total NMR and NMR due to non‐CA causes. Similarly, no time trend was noted in the rate of neonatal deaths attributable to CA. Comparing the total NMR reported from 1994 to 2013 (5.2 per 1000 live births) and the total NMR found in this study (4.4 per 1000 live births) it is seen that the total NMR has remained stable and is within the required target value proposed by SDG 3.2.2.^2^, ^13^

Unlike studies of other EU countries, in this study education was not found to be a driver for NMR and no educational inequalities in neonatal mortality were observed.^8^ While this could be due to missing data, it could also be due to less variation in education levels reported in Malta. Another explanation is that healthcare is equally accessible and free at point of contact for all residents across all income levels, accessibility is typically related to educational level.^8^ According to EUROSTAT data on self‐reported unmet needs for medical examination among females aged 16–44 and categorised by income quintile in Malta, it was demonstrated that within this group, there existed minimal inequality in access to healthcare. This was evident from a difference of only 0.10 between the first and fifth quintile, whereas the EU average showed a difference of 2.40.^21^, ^22^

Over the past years, Malta has experienced a high influx of migration, with the number of foreigners more than quintupling since 2011.^15^ Since 2013, when data became available for all EU member states, most countries in the EU have shown an increase in live births from foreign‐born mothers. According to EUROSTAT's data, Malta experienced the highest increase of live births in foreign‐born mothers, rising by 22 percentage points (pp) from 11% in 2013 to 33% in 2022. Portugal followed with an 8 pp increase, from 24% to 16%, while Spain, Cyprus, and Slovenia all saw increases of 7 pp.^23^

Several studies, including studies from Malta, indicate that the risk of adverse neonatal outcomes is higher in migrants when compared with native‐origin women,^22^, ^24^, ^25^ despite well‐functioning healthcare.^9^, ^26^ A study by Bollini et al.^9^ shows that in European countries, the risk for neonatal mortality is higher for immigrant women, mostly due to inadequate prenatal care. They also face higher risks of stillbirth and CA compared to indigenous women, indicating underlying mechanisms beyond migration and socioeconomics. To address these disparities, understanding risk factors across different migrant groups is crucial, requiring tailored antenatal care, early screening, and improved health literacy among women and healthcare professionals.^27^, ^28^ Efforts to improve care of migrant mothers in Malta has led to the establishment of a Migrant Health Liaison Office in 2008.^29^ Nevertheless, Maltese midwives and obstetricians still report challenges in communication, compliance to antenatal appointments and treatments.^25^ Women born in Sub‐Saharan Africa (SSA), have been reported to be at higher risk due to their socioeconomic status, health condition and cultural attitudes towards obstetric care.^22^, ^24^ They are less likely to utilise available obstetric care and social support services.^22^ Also, multiparty in SSA women was found to be associated with a higher rate of neonatal death.^22^, ^24^ The high representation of women from Somalia in Malta, who have a fertility rate of 7.0 as described by Hili et al.^22^ could contribute to the relatively higher neonatal mortality. Additionally, the increasing presence of mothers from conflict zones including Syria and Eritrea could contribute to neonatal mortality related to their immigrant background and challenging health conditions.^26^, ^29^

A time trend analysis conducted from 1994 to 2015 highlighted that the majority of EU countries displayed evidence of downwards trends in NMR.^30^ The higher rate of neonatal mortality related to CA causes in Malta, is likely to be related to TOPFA being illegal.^13^ In this study the highest NMR occurred during the early neonatal period. The high NMR at an early stage may be related to the births of infants with fatal CA, who would have been terminated in other countries but are carried to term in Malta, resulting in their passing shortly after birth and being included in NMR. This contributes to the overall NMR in Malta. It is also notable that in spite of countries practising TOPFA, there are still inter‐country variations in the accessibility and uptake of such a service.^31^, ^32^

The contribution of non‐CA causes to the total NMR in Malta was the third highest among other EU countries. It is evident that countries that have been facing challenges in the past, have made more substantial progress in reducing neonatal deaths due to non‐CA causes, such as Romania and Poland where there has been greatly improved paediatric and neonatal care.^33^ In comparison to the EU average, Malta's progress has been shown to be slower, possibly due to demographic changes in maternal nationalities which have been shown to influence NMR also in this current study.

Neonatal survival across European countries has decreased, however it displays marked variation across countries and gestational age. This is affected by perinatal decision‐making and varying management guidelines for extreme preterm births. Regional and hospital‐level differences further contribute to variability in perinatal survival outcomes. Disparities in delivery of care for extremely preterm births influence morbidity and mortality outcomes as do improvements in obstetric care, modifications in the timing, recipients and methods of administering therapies like steroids, surfactant and emerging treatments such as antenatal magnesium sulphate, delayed cord clamping, and placental transfusion.^34^, ^35^, ^36^

This study has shown Malta (where TOPFA is illegal) to have a higher NMR due to CA than other EU countries. It has also highlighted differences in NMR associated with maternal nationality. Antenatal screening practices also influence NMR, since TOPFA is not an option in Malta, routine antenatal screening of uncomplicated pregnancies is limited to two ultrasounds at 10–13 weeks (dating scan) and 28–32 weeks (growth scan) and one anomaly scan at 19–23 weeks taken for purposes of management of delivery. Further investigations are only done at maternal request in the private sector. Other possible factors which may influence NMR but were beyond the scope of this study include, gestational age at birth and approaches to survival focused care and facilities, especially for extremely preterm babies.^35^, ^36^, ^37^ These parameters merit further research to fully understand and interpret NMRs and identify key issues for intervention.

## 5 CONCLUSIONS

In conclusion, the overall NMR of Malta did not change between 2006 and 2020, with 39.6% of neonatal mortality attributed to CA. When adjusting NMR for maternal education, nationality, and age, only nationality was found as being associated with a higher risk of neonatal mortality, with women from non‐EU, low‐income countries being more likely to be affected. Comparing to other EU countries, Malta continues to register the highest NMR reported in the EU. This is in part due to the proportionally higher contribution of neonatal mortality attributed to CA causes, which in turn is partly influenced by TOPFA being illegal. The situation presents a public health challenge, especially considering the rapidly changing demography with increasing immigration of women from low‐income countries. These factors necessitate continuing research and implementation of monitored, directed and effective primary prevention strategies. Exploration of other factors potentially influencing NMR including antenatal screening methods for CA and neonatal healthcare practices are important avenues for future research.

## AUTHOR CONTRIBUTIONS

The study was conceived, and the initial draft was written by MW. Further drafts were reviewed, commented on and revised by MG, RH, NC, and HZ. MW conducted the data analysis and directly accessed and verified the underlying data reported in the study.

## CONFLICT OF INTEREST STATEMENT

None.

## Supporting information


Data S1:

## Data Availability

The data that support the findings of this study are available on request from the corresponding author. The data are not publicly available due to privacy or ethical restrictions.
