# Supplementary material for: Evaluating neonatal mortality in Malta compared with other EU countries: Exploring the influence of congenital anomalies and maternal risk factors
Source: Paediatr Perinat Epidemiol. 2024 Sep 6;38(8):703–13. doi: 10.1111/ppe.13106 (PMC11603760; doi:10.1111/ppe.13106)
Supplement: Supplementary file 1 — Data S1: [file PPE-38-703-s001.zip › Flow Chart.docx]

**Flow Chart:**

Analysed 63890 live births

of which 283 were neonatal deaths (230 early and 53 late neonatal death) and the rest lived beyond the neonatal period.

Births data from the years 2006 – 2020 extracted (*n* = 63,890 births)

321 stillbirths excluded

Total exclusions (*n* = 321)
