# Supplementary material for: Evaluating neonatal mortality in Malta compared with other EU countries: Exploring the influence of congenital anomalies and maternal risk factors
Source: Paediatr Perinat Epidemiol. 2024 Sep 6;38(8):703–13. doi: 10.1111/ppe.13106 (PMC11603760; doi:10.1111/ppe.13106)
Supplement: Supplementary file 1 — Data S1: [file PPE-38-703-s001.zip › Supplementary material _1.docx]

**Supplementary material**

**Table of contents**

[**eTable 1** Descriptive statistics for maternal education, nationality and age from 2006-2020 in Malta 1](#_Toc162168931)

[**eTable 2** Results of the Logistic regression on the impact of maternal education, nationality and age on NMR in Malta from 2006-2020 2](#_Toc162168932)

**eTable 1 Descriptive statistics for maternal education, nationality and age from 2006-2020 in Malta**

| **Characteristic** |  | **Category** | **Frequency** | **Percent** |
| --- | --- | --- | --- | --- |
| **Education Level** |  | Non tertiary education | 31135 | 48·7% |
|  |  | Tertiary education | 18620 | 29·1% |
|  |  | Missing | 14135 | 22·1% |
|  |  | Total | 63890 | 100·0% |
| **Age** |  | < 19 | 2839 | 4·4% |
|  |  | 20-35 | 52443 | 82·1% |
|  |  | > 35 | 8608 | 13·5% |
|  |  | Missing | 24 | 0·04% |
|  |  | Total | 63890 | 100·0% |
| **Nationality** |  | Maltese | 53249 | 83·3% |
|  |  | Pre2004 EU | 2343 | 3·7% |
|  |  | Post2004 EU | 1667 | 2·6% |
|  |  | NE high income | 263 | 0·4% |
|  |  | NE upper middle income | 2643 | 4·1% |
|  |  | NE lower middle income | 1890 | 3·0% |
|  |  | NE low income | 1657 | 2·6% |
|  |  | Missing | 178 | 0·3% |
|  |  | Total | 63890 | 100·0% |
| **Infant Outcome** |  | Death | 283 | 0·4% |
|  |  | Alive | 63607 | 99·6% |
|  |  | Total | 63890 | 100·0% |
| **Cause of Death** |  | Congenital | 99 | 0·2% |
|  |  | Non-congenital | 151 | 0·2% |
|  |  | Alive | 63607 | 99·6% |
|  |  | Missing  Total | 33  63890 | 0·1%  100·0% |

NE=non-EU

**eTable 2 Results of the Logistic regression on the impact of maternal education, nationality and age on NMR in Malta from 2006-2020**

| **Characteristics** | **Tests of Model Effects:** | **Categories** | **OR** | **95% CI** | **p-Value** |
| --- | --- | --- | --- | --- | --- |
| **Nationality** | 0·025 | Maltese | 1 | ·· | ·· |
|  |  | Pre2004 EU | 0·98 | 0·52, 1·85 | 0·95 |
|  |  | Post2004 EU | 0·69 | 0·28, 1·67 | 0·41 |
|  |  | NE high income | 2·64 | 0·84, 8·29 | 0·10 |
|  |  | NE upper middle income | 1·04 | 0·58, 1·86 | 0·89 |
|  |  | NE lower middle income | 0·61 | 0·25, 1·47 | 0·27 |
|  |  | NE low income | 2·23 | 1·34, 3·71 | 0·00 |
| **Age** | 0·100 | <19 | 1·18 | 1·18, 0·69 | 0·55 |
|  |  | 20-35 | 1 | ·· | ·· |
|  |  | >35 | 1·39 | 1·02, 1·90 | 0·04 |
| **Education** | 0·152 | Non tertiary education | 1·24 | 0·92, 1·68 | 0·15 |
|  |  | Tertiary Education | 1 | ·· | ·· |

NE= non-EU
