# Supplementary material for: Evaluating neonatal mortality in Malta compared with other EU countries: Exploring the influence of congenital anomalies and maternal risk factors
Source: Paediatr Perinat Epidemiol. 2024 Sep 6;38(8):703–13. doi: 10.1111/ppe.13106 (PMC11603760; doi:10.1111/ppe.13106)
Supplement: Supplementary file 1 — Data S1: [file PPE-38-703-s001.zip › Supplementary material _2.docx]

**Supplementary material**

**Table of contents**

[**eTable 1** Descriptive statistics for maternal education, nationality and age from 2006-2020 in Malta 1](#_Toc162168931)

[**eTable 2** Results of the Logistic regression on the impact of maternal education, nationality and age on NMR in Malta from 2006-2020 2](#_Toc162168932)

**eTable 1 Descriptive statistics for maternal education, nationality and age from 2006-2020 in Malta**

| **Characteristic** |  | **Category** | **Frequency** | **Percent** |
| --- | --- | --- | --- | --- |
| **Education Level** |  | Non tertiary education | 31135 | 48.7% |
|  |  | Tertiary education | 18620 | 29.1% |
|  |  | Missing | 14135 | 22.1% |
|  |  | Total | 63890 | 100.0% |
| **Age** |  | < 19 | 2839 | 4.4% |
|  |  | 20-35 | 52443 | 82.1% |
|  |  | > 35 | 8608 | 13.5% |
|  |  | Missing | 24 | 0.04% |
|  |  | Total | 63890 | 100.0% |
| **Nationality** |  | Maltese | 53249 | 83.3% |
|  |  | Pre2004 EU | 2343 | 3.7% |
|  |  | Post2004 EU | 1667 | 2.6% |
|  |  | NE high income | 263 | 0.4% |
|  |  | NE upper middle income | 2643 | 4.1% |
|  |  | NE lower middle income | 1890 | 3.0% |
|  |  | NE low income | 1657 | 2.6% |
|  |  | Missing | 178 | 0.3% |
|  |  | Total | 63890 | 100.0% |
| **Infant Outcome** |  | Death | 283 | 0.4% |
|  |  | Alive | 63607 | 99.6% |
|  |  | Total | 63890 | 100.0% |
| **Cause of Death** |  | Congenital | 99 | 0.2% |
|  |  | Non-congenital | 151 | 0.2% |
|  |  | Alive | 63607 | 99.6% |
|  |  | Missing  Total | 33  63890 | 0.1%  100.0% |

NE=non-EU

**eTable 2 Results of the Logistic regression on the impact of maternal education, nationality and age on NMR in Malta from 2006-2020**

| **Characteristics** | **Tests of Model Effects:** | **Categories** | **OR** | **95% CI** | **p-Value** |
| --- | --- | --- | --- | --- | --- |
| **Nationality** | 0.025 | Maltese | 1 | .. | .. |
|  |  | Pre2004 EU | 0.98 | 0.52, 1.85 | 0.95 |
|  |  | Post2004 EU | 0.69 | 0.28, 1.67 | 0.41 |
|  |  | NE high income | 2.64 | 0.84, 8.29 | 0.10 |
|  |  | NE upper middle income | 1.04 | 0.58, 1.86 | 0.89 |
|  |  | NE lower middle income | 0.61 | 0.25, 1.47 | 0.27 |
|  |  | NE low income | 2.23 | 1.34, 3.71 | 0.00 |
| **Age** | 0.100 | <19 | 1.18 | 0.69, 2.03 | 0.55 |
|  |  | 20-35 | 1 | .. | .. |
|  |  | >35 | 1.39 | 1.02, 1.90 | 0.04 |
| **Education** | 0.152 | Non tertiary education | 1.24 | 0.92, 1.68 | 0.15 |
|  |  | Tertiary Education | 1 | .. | .. |

NE= non-EU
